# The role of iliopsoas fractional lengthening in hip arthroscopy: a systematic review

**DOI:** 10.1093/jhps/hnad039

**Published:** 2023-11-15

**Authors:** Alexander Baur, Wesley Lemons, James Satalich, Alexander Vap, Robert O’Connell

**Affiliations:** 2nd year Medical Student, Liberty University College of Osteopathic Medicine, Lynchburg, VA 24502, USA; PGY-1, Virginia Commonwealth University School of Medicine, Richmond, VA 23298, USA; PGY-4, Virginia Commonwealth University School of Medicine, Richmond, VA 23298, USA; Department of Orthopaedic Surgery, Virginia Commonwealth University School of Medicine, Richmond, VA 23298, USA; Department of Orthopaedic Surgery, Virginia Commonwealth University School of Medicine, Richmond, VA 23298, USA

## Abstract

Arthroscopic iliopsoas fractional lengthening (IFL) is a surgical option for the treatment of internal snapping hip syndrome (ISHS) after failing conservative management. Systematic review. A search of PubMed central, National Library of Medicine (MEDLINE) and Scopus databases were performed by two individuals from the date of inception to April 2023. Inclusion criteria were ISHS treated with arthroscopy. Sample size, patient-reported outcomes and complications were recorded for 24 selected papers. Preferred Reporting Items for Systematic Reviews and Meta-Analyses guidelines were followed and registered on PROSPERO database for systematic reviews (CRD42023427466). Thirteen retrospective case series, ten retrospective comparative studies, and one randomized control trial from 2005 to 2022 were reported on 1021 patients who received an iliopsoas fractional lengthening. The extracted data included patient satisfaction, visual analogue scale, the modified Harris hip score and additional outcome measures. All 24 papers reported statistically significant improvements in post-operative patient-reported outcome measures after primary hip arthroscopy and iliopsoas fractional lengthening. However, none of the comparative studies found a statistical benefit in performing IFL. Existing studies lack conclusive evidence on the benefits of Iliopsoas Fractional Lengthening (IFL), especially for competitive athletes, individuals with Femoroacetabular Impingement (FAI), and borderline hip dysplasia. Some research suggests IFL may be a safe addition to hip arthroscopy for Internal Snapping Hip Syndrome, but more comprehensive investigations are needed. Future studies should distinguish between concurrent procedures and develop methods to determine if the psoas muscle is the source of pain, instead of solely attributing it to the joint.

## INTRODUCTION

Hip pain is a common orthopedic condition that can significantly impact an individual’s quality of life [1]. Internal snapping hip syndrome (ISHS), which presents with a snapping sensation over the hip joint during certain activities, can be a cause of hip pain. The condition occurs when the iliopsoas tendon slides over the iliopectinal eminence or the anterior aspect of the femoral head. Oftentimes, this mechanism can lead to iliopsoas tendinitis. While conservative measures are often used for initial management, surgical intervention may be necessary if these methods fail [2].

Arthroscopic iliopsoas release is a minimally invasive surgical option that involves releasing the iliopsoas tendon to reduce friction and snapping in the hip joint [3]. Despite the well-defined arthroscopic techniques, there still remain several controversial aspects of the procedure. The release of the iliopsoas carries inherent risks and has the potential to heighten instability or lead to intraabdominal fluid extravasation [3]. Surgeons often run into the question of whether to combine iliopsoas releases with their arthroscopic labral repairs when a patient has a concomitant internal snapping hip pathology. In addition, iliopsoas impingement may be found during arthroscopy and there is no consensus of whether to treat the impingement in addition to the original labral pathology [4].

To inform clinical decision-making, this systematic review examines the efficacy and safety of performing an iliopsoas release during hip arthroscopy. The studies reviewed provide insights into the medium and long-term functional outcomes, return to sport rates, and reoccurrence of hip pain following surgical intervention. The results of this systematic review will help optimize patient outcomes in the management of internal snapping hip.

## METHODS

This systematic review adhered to the Preferred Reporting Items for Systematic Reviews and Meta-Analyses guidelines to identify a final selection of papers for analysis. The plan was registered on PROSPERO before starting the systematic review (CRD42023427466). The inclusion criteria for this study specified patients diagnosed with ISHS who underwent arthroscopic iliopsoas fractional lengthening (IFL). Exclusion criteria encompassed open surgical procedures, previous total hip arthroplasty and studies lacking patient outcome reporting.

To compile the definitive list of papers for inclusion, a comprehensive search was conducted of the PUBMED Central, MEDLINE, and Scopus databases by two independent reviewers. The search strategy employed the following Boolean terms: ‘iliopsoas OR snapping hip OR extra-articular hip’ in the title, resulting in an initial pool of 1103 papers. Subsequently, the inclusion criteria were refined by incorporating ‘arthroscopy OR surgical management’ in any field, reducing the number to 176 papers.

Further application of exclusion criteria involved excluding papers with the keywords ‘arthroplasty OR external snapping’ in the title, which yielded a set of 75 papers. The abstracts of these papers were then scrutinized, leading to the exclusion of various study types such as cadaveric studies, surgical tutorials, commentary, case studies, imaging studies and systematic reviews. Additional exclusion criteria were employed based on the subject matter of the study, resulting in the elimination of papers related to abscesses, hematomas and studies with unreported patient outcome measures. Ultimately, a total of 17 papers met the inclusion and exclusion criteria and were reviewed and analyzed to address the research question of this study.

**Fig. 1.**
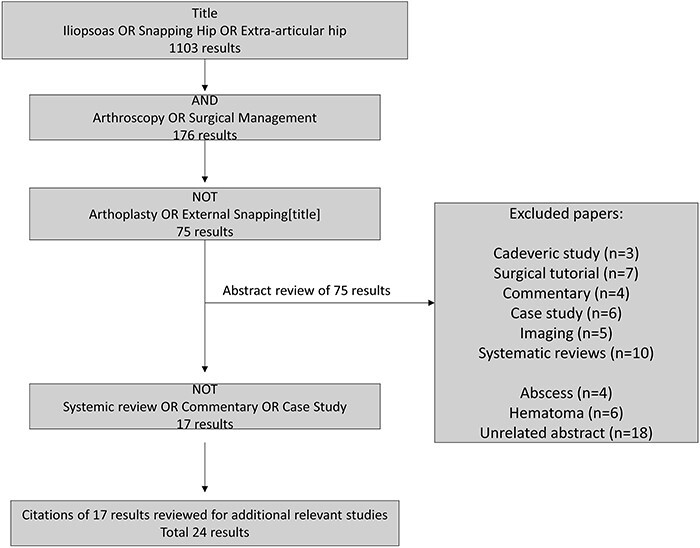
Methodology flow chart.

To augment the analysis, the references of these 17 papers were examined, leading to the identification of 7 additional relevant studies. Consequently, a final set of 24 papers was utilized for the comprehensive analysis of the research question Figure 1.

## RESULTS

### Paper characteristics

Thirteen papers from 2005 to 2018 reported on case series with cohort sizes ranging from 6 to 67, totaling 374 patients. An additional nine papers from 2016 to 2022 reported retrospective comparative studies with a total of 641 patients in the study groups and 704 patients in the control groups. Additionally, one randomized controlled trial in 2009 and a similar comparative study in 2014 reported on smaller patient groups of two different techniques, with 10 and 9 patients in one and 6 and 14 patients in the other, respectively. Various surgical techniques were examined and listed in Table I. Eighteen papers reported a modified Harris Hip Score (mHHS), ten papers reported a visual analogue scale (VAS), eight papers reported a non-arthritic hip score (NAHS), five papers reported a hip outcome score (HOS), five papers reported a hip outcome score sport scale (HOS-SSS), four papers reported a hip outcome score activities daily living (HOS-ADL), five papers reported patient satisfaction, four papers reported an international hip outcome tool (iHOT) and 3 papers reported a Western Ontario and McMaster Universities Osteoarthritis Index (WOMAC).

**Table I.** Study design, sample size and outcome scores

*Study (Authors)*	*Study design*	*Sample size*	*Outcome scores*		
Ilizaliturri *et al*. [2005] [14]	Prospective case series [4]	6 patients and 7 hips	WOMAC score:		
			Pre-operative 82.5 Post-operative 91		
Flanum *et al*. [2007] [15]	Retrospective case series [4]	6 patients	mHHS:		
			Pre-operative 58.3 6 weeks post-operative 62.3 12 weeks post-operative 84.5 24 weeks post-operative 90.3 1 year post-operative 95.7		
Anderson and Keene [2008] [16]	Retrospective case series [4]	15 athletes (5 competitive and 10 recreational)	mHHS:		
			Pre-operative recreational 44.4 52 weeks recreational 96.0 Pre-operative competitive 40.6 52 weeks competitive 96.8		
Ilizaliturri *et al*. [2009] [17]	Randomized control trial	19 patients	WOMAC score:		
			Group 1 (iliopsoas release at lesser trochanter)	Group 2 (Transcapsular iliopsoas tendon release)	
			Pre-operative 70.1 1 year post-operative 83.7	Pre-operative 67 1 year post-operative 83.6	
Conteras *et al*. [2010] [18]	Retrospective case series [4]	7 patients	mHHS:		
			Pre-operative 56.1 12 months post-operative 88.4 (*P*-value 0.018) 24 months post-operative 87.9 (*P*-value 0.02)		
			VAS:		
			Pre-operative 7.7 3 months post-operative 4.3 (*P*-value 0.051) 6 months post-operative 3.6 (*P*-value 0.015) 12 months post-operative 2.4 (*P*-value 0.011) 24 months post-operative 2.4 (*P*-value 0.011)		
Domb *et al*. [2011] [19]	Retrospective case series [4]	25 patients	mHHS:		
			Post-operative 87.17		
			HOS-SSS:		
			Post-operative 78.8		
			HOS-ADL:		
			Post-operative 92.46		
Fabricant *et al*. [2012] [20]	Retrospective case series [4]	67 patients	Low/normal version	High version	
			mHHS:		
			Pre-operatively 61.3 ± 16.3 Post-operatively 86.1 ± 14.8	Pre-operatively 66.0± 13.5 Post-operatively 76.9± 16.8	
			*P*-value 0.031 between two groups post-operatively		
			HOS-ADL:		
			Pre-operative 69.6 ± 18.2	Pre-operative 66.0 ± 11.9	
			Post-operative 87.9 ± 14.4	Post-operative 82.5 ± 18.2	
			HOS-SSS		
			Pre-operative 50.0± 24.7	Pre-operative 26.6 ± 21.5	
			Post-operative 70.7 ± 25.6	Post-operative 59.4 ± 33.7	
Hain *et al*. [2013] [21]	Retrospective case series [4]	20 patients	mHHS:		
			79		
Garala *et al*. [2014] [22]	Retrospective case series [4]	15 patients	NAHS:		
			Post-operative 66.4		
Ilizaliturri *et al*. [2014] [23]	Prospective comparative study [2]	20 patients	Central release	Lesser trochanter release	
			WOMAC score:		
			Pre-operative 56 ± 13.21	Pre-operative 46.33 ± 21.83	
			Post-operative 89.57 ± 3	Post-operative 89.33 ± 1.36	
El Bitar *et al*. [2014] [24]	Prospective case series [4]	55 patients	mHHS:		
			Pre-operatively 63.3 ± 17.5		
			2-year follow-up 84.6 ± 16.5		
			*P*-value <0.001		
			VAS:		
			Pre-operatively 6.0 ± 2.2		
			2-year follow-up 2.4 ± 2.1		
			*P*-value <0.001		
			HOS-SSS:		
			Pre-operatively 43.8 ± 25.9		
			2-year follow-up 74.4 ± 24.4		
			HOS-ADL		
			Pre-operatively 61.9 ± 22.0		
			2-year follow-up 85.5 ± 18.4		
			*P*-value <0.001		
			NAHS:		
			Pre-operatively 58.2 ± 20.3		
			2-year follow-up 84.1 ± 16.5		
			*P*-value <0.001		
Nelson and Keene [2014] [12]	Retrospective case series [4]	30 patients	mHHS:		
			Pre-operative 43		
			6-week post-operative 71		
			6-months post-operative 81		
			1-year post-operative 84		
Hwang *et al*. [2015] [25]	Retrospective case series [4]	25 patients	mHHS:		
			Pre-operative 65		
			Post-operative 84		
			*P*-value <0.0001		
			VAS:		
			Pre-operative 6		
			Post-operative 2		
			HOS-SSS:		
			Pre-operative 60%		
			Post-operative 82%		
			HOS-ADL:		
			Pre-operative 66%		
			Post-operative 87%		
Brandenburg *et al*. [2016] [26]	Retrospective comparative study [3]	2 groups of 18 patients	NR		
Mardones *et al*. [2016] [27]	Retrospective case series [4]	15 patients	mHHS:		
			Pre-operative 74.7		
			Post-operative 95.8		
			VAS:		
			Pre-operative 5.5		
			Post-operative 0		
Walczak *et al*. [2017] [9]	Prospective case series [4]	28 patients	mHHS:		
			Group 1 (atrophy of grades 4,3 and 2)	Group 2 (Atrophy of grades 1 and 0)	
			Pre-operative 48.0	Pre-operative 42.7	
			Score at 2nd MRA 70.5	Score at 2nd MRA 64.1	
Hartigan *et al*. [2018] [28]	Retrospective case series [4]	32 patients	mHHS:		
			Pre-operative 68.7		
			Latest 83.5		
			*P*-value <0.001		
			VAS:		
			Pre-operative 5.6		
			Latest 1.9		
			NAHS:		
			Pre-operative 64.9		
			Latest 86.8		
			*P*-value <0.001		
			HOS-ADL:		
			Pre-operative ADL 71.6		
			Latest ADL 86.7		
			*P*-value ADL <0.001		
			HOS-SSS:		
			Pre-operative SSS 52.6		
			Latest SSS 75.8		
			*P*-value SSS <0.001		
Perets *et al*. [2018] [6]	Retrospective comparative study [3]	60 patients	IFL group	Control group (Athletes not requiring IFL)	
			mHHS:		
			Pre-operative 65.7 ± 12.1	No significant differences	
			latest 82.4 ± 14.1	No significant differences	
			*P*-value <0.001	No significant differences	
			VAS:		
			Pre-operative 5.7 ± 2.3	No significant differences	
			latest 2.6 ± 2.4	No significant differences	
			*P*-value <0.001	No significant differences	
			HOS:		
			Pre-operative 44.1 ± 17.7	No significant differences	
			latest 73.0 ± 24.7	No significant differences	
			*P*-value <0.001	No significant differences	
			NAHS:		
			Pre-operative 64.2 ± 16.6	No significant differences	
			latest 83.2 ± 15.8	No significant differences	
			*P*-value <0.001	No significant differences	
			Patient satisfaction		
			7.9 ± 1.9		
Maldonado *et al*. [2018] [29]	Retrospective comparative study [3]	351 hips IFL and 392 hips in control	IFL group	Control group (Without IFL)	*P*-value
			mHHS:		
			83.2 ± 15.8	84.0 ± 16.8	0.24
			HOS:		
			72.1 ± 26.6	73.3 ± 27.1	0.69
			iHot		
			71.4 ± 25.9	72.2 ± 26.1	0.41
Perets *et al*. [2019] [7]	Retrospective comparative study [3]	57 patients in both IFL and control group	IFL group	Control group	
			mHHS:		
			Pre-operative 64.3 ± 13.6 Latest 84.9 ± 15.8 *P*-value <0.001	Pre-operative 61.6 ± 14.4 Latest 85.9 ± 13.5 *P*-value <0.001	0.298 0.907
			VAS:		
			Pre-operative 6.5 ± 2.1 Latest 2.2 ± 2.0 *P*-value <0.001	Pre-operative 5.8 ± 2.1 Latest 2.3 ± 2.3 *P*-value <0.001	0.171 0.965
			HOS		
			Pre-operative 47.0 ± 21.6 Latest 75.0 ± 24.0 *P*-value <0.001	Pre-operative 45.9 ± 22.9 Latest 75.9 ± 20.8 *P*-value <0.001	0.784 0.859
			NAHS		
			Pre-operative 61.7 ± 18.2 Latest 85.2 ± 15.7 *P*-value <0.001	Pre-operative 65.14 ± 15.75 Latest 82.96 ± 17.97	0.436 0.576
			Patient satisfaction		
			8.1 ± 1.7	8.2 ± 1.6	0.835
Meghpara *et al*. [2020] [4]	Retrospective comparative study [3]	37 hips in non-IFL group and 87 hips in IFL group	IFL group	Control group	
			mHHS:		
			Pre-operative 63.61 ± 16.15 Latest 86.10 ± 16.45	Pre-operative 63.87 ± 13.06 Latest 86.06 ± 15.31	0.53
			VAS:		
			Pre-operative 4.95 ± 2.40 Latest 2.19 ± 2.61	Pre-operative 5.49 ± 2.28 Latest 2.24 ± 2.28	
			HOS		
			Pre-operative 41.35 ± 23.31 Latest 76.54 ± 25.52	Pre-operative 39.96 ± 19.26 Latest 78.14 ± 20.67	0.87
			NAHS:		
			Pre-operative 62.51 ± 18.18 Latest 84.73 ± 17.31	Pre-operative 65.14 ± 15.75 Latest 82.96 ± 17.97	0.40
			iHOT		
			77.54 ± 24.48	74.47 ± 21.33	
			Patient satisfaction		
			8.01 ± 2.50	8.47 ± 1.83	
Maldonado *et al*. [2021] [8]	Retrospective comparative study [3]	74 hips (29 matched 1:1)	IFL group	Control group	
			mHHS:		
			Pre-operative 59.24 ± 14.75 Latest 79.79 ± 19.46 *P*-value <0.0001	Pre-operative 62.26 ± 13.41 Latest 87.12 ± 14.63 *P*-value <0.0001	0.418 0.141
			VAS:		
			Pre-operative 5.54 ± 2.38 Latest 3.19 ± 2.65 *P*-value 0.0005	Pre-operative 4.84 ± 2.18 Latest 1.99 ± 1.98 *P*-value <0.0001	0.345 0.058
			HOS		
			Pre-operative 43.86 ± 26.07 Latest 66.18 ± 29.62 *P*-value 0.0004	Pre-operative 39.86 ± 20.82 Latest 70.52 ± 24.06 *P*-value <0.0001	0.528 0.065
			NAHS		
			Pre-operative 59.37 ± 18.91 (52.49–66.25) Latest 78.02 ± 22.96 (68.90–85.66) *P*-value <0.0001	Pre-operative 61.91 ± 16.55 (55.88–67.93) Latest 87.27 ± 12.39 (82.59–91.94) *P*-value <0.0001	0.589 0.065
			iHOT		
			67.23 ± 27.52 (57.21–77.24)	77.72 ± 19.62 (70.17–85.26)	0.199
			Patient satisfaction		
			7.14 ± 3.32 (5.93–8.35)	8.42 ± 1.88 (7.70–9.15)	0.081
Matsuda *et al*. [2021] [10]	Retrospective comparative study [3]	16 patients (compared to 76 who did not undergo surgery for iliopsoas)	IFL group	Control group	
			iHOT		
Pre-operative 35 [24] Post-operative 57 [11]			Pre-operative psoas involvement without tenotomy 36 [27] Post-operative psoas involvement without tenotomy 67(32)	Pre-operative without psoas involvement 35 [26] Post-operative without psoas involvement 73 [7]	0.95 0.02
Jimenez *et al*. [2022] [5]	Retrospective comparative study [3]	42 athletes in IFL group and 54 matched control athletes	IFL group	Control group	
			mHHS:		
			Pre-operative 64.2 ± 13.8 Latest 88.4 ± 14.2 *P*-value <0.001	Pre-operative 67.0 ± 14.7 (33.0 to 96.0) Lastest 87.9 ± 15.7 *P-*value <0.001	0.360 0.789
			VAS:		
			Pre-operative 5.0 ± 2.4 Latest 2.1 ± 2.2 *P*-value <0.001 Improvement 3.3 6 3.3	Pre-operative 4.9 ± 2.8 Latest 1.9 ± 2.3 *P*-value <0.001	0.892 0.466
			HOS:		
			Pre-operative 44.6 ± 20.0 Latest 79.4 ± 26.1 *P*-value <0.001	Pre-operative 51.5 ± 23.8 Latest 81.1 ± 22.5 *P*-value <0.001	0.137 0.795
			NAHS:		
			Pre-operative 63.1 ± 16.6 Latest 87.0 ± 15.5 *P*-value <0.001	Pre-operative 68.8 ± 19.8 Latest 87.2 ± 15.9) *P*-value <0.001	0.052 0.899
			Patient satisfaction		
			Patient satisfaction 8.6 ±1.7	Patient Satisfaction 8.1 ± 2.3	0.238

All papers reported primarily on the outcomes following IFL or iliopsoas tenotomy but included various concomitant arthroscopic repairs as well. While the primary focus of the papers was the IFL, additional procedures listed included debridement, acetabuloplasty, femoroplasty, labral repair and capsule repair.

### Clinical outcomes

This systematic review provides evidence for the effectiveness of surgical treatments for ISHS, particularly IFL, in alleviating associated symptoms. Patients who underwent IFL in conjugation with other surgical interventions reported improved patient-reported outcome (PRO) scores, pain and snapping resolution and restoration of function. The results of this systematic review provide important clinical considerations for patients undergoing hip arthroscopy with concomitant internal snapping hip such as athletes, patients with femoroacetabular impingement (FAI) and patients with hip dysplasia. In addition, new comparative studies question whether IFL is beneficial for all patients with ISHS.

When looking at athletes, two recent studies by Jimenez *et al*. and Perets *et al*. evaluate the use of IFL and the impact on return to sport [5, 6]. In the Jimenez *et al*. study, athletes with FAI and painful ISHS were identified as a specific patient population that could benefit from intrabursal IFL [5]. The study found that this treatment approach was effective for managing hip pain in these athletes, allowing them to return to their pre-injury level of sport and achieve favorable functional outcomes. The Perets *et al*. study, on the other hand, focused on athletes with hip flexor pain and found that arthroscopic IFL can be an effective treatment option for this population [6]. These studies suggest that IFL can be used in conjunction with arthroscopic labral repair surgery to manage hip pain in competitive athletes.

Another large population undergoing hip arthroscopy are patients with labral tears and FAI. The Perets and Maldonado study both added IFL to this population. Both of these studies found IFL does not adversely affect clinical outcomes [7, 8]. However, there was no statistical significant improvement in patient outcomes.

Similar to FAI, hip dysplasia is a condition that can affect individuals with ISHS, especially women. It is a condition where the socket of the hip joint is slightly shallow, which can lead to early degeneration of the hip joint and other hip pathologies. Importantly, the degree of hip dysplasia should be carefully considered prior to surgery. In patients with moderate to severe hip dysplasia, arthroscopic treatment may not be sufficient and they may require a periacetabular osteotomy. A recent study by Maldonado *et al*. focused on the addition of IFL with hip arthroscopy in female patients with borderline dysplasia and painful ISHS. In this population, iatrogenic hip instability is of increased concern because the iliopsoas is an important dynamic anterior stabilizer. In their study, Maldonado performed the IFL with capsular plication to address this instability risk. The Jimenez *et al*. study reiterated the importance of capsular management in individuals at risk of instability. In their study, Maldonado *et al*. study found that IFL followed by capsular plication was beneficial to patients in addition to a primary arthroscopic hip procedure. Specifically, the paper found significantly improved patient outcome measures and there were no instances of post-operative complications or reoperations related to IFL. Even with this paper showing positive results, this procedure is highly controversial due to the inherent risk of instability.

While there is positive evidence for IFL, it is important not to overlook the potential complications associated with this procedure. IFL and tenotomy have been linked to damaging surrounding soft tissues, such as the iliopsoas and gluteal tendons, or the lateral femoral cutaneous nerve, according to Walczak *et al*. [9]. Iliopsoas tendon tears are a common complication of IFL arthroscopy, with some studies reporting rates as high as 20%. Revision surgery and conversion to total hip arthroplasty (THA) may also be necessary in some cases. Additionally, two recent studies by Matsuda *et al*. (2021) and Meghpara *et al*. (2020) have highlighted the importance of careful patient selection and judicious use of IFL [4, 10]. Matsuda *et al*. found that only 1% of their patients undergoing arthroscopic hip surgery had iliopsoas pathology, and only 17% of those with pathology were surgically managed [10]. Meghpara *et al*. found that IFL did not significantly improve PRO for patients without painful internal snapping, further emphasizing the need for careful patient selection and consideration of alternative treatment options [4]. Thus, iliopsoas tenotomy and IFL should not be routinely used without clear internal snapping hip pathology present pre-operatively.

## DISCUSSION

This literature review builds on the findings of two previous studies by Gouveia *et al*. and Longstaffe *et al*. in 2021 regarding the effectiveness of arthroscopic release of the iliopsoas tendon (IFL) for the treatment of ISHS [3, 11]. Since the cutoff for these systematic reviews in 2018, six additional level 3 comparative cohort studies have provided significant clinical insights into the use of IFL. Both review papers found that the procedure is an effective treatment for internal snapping hip. However, prior to 2020, no paper focused on IFL concluded that the procedure should be used with caution. Before the Meghpara *et al*. paper in 2020, all the papers reviewed in this paper emphasized the benefits of IFL and the positive PRO results [4]. However, since the pioneering paper in 2005, many complications have been reported. Table II in this review discusses the complications each paper reported, including the Meghpara *et al*. paper and a paper by Matsuda *et al*., which directly reported negative outcomes for the IFL group in their study [4, 10]. An important complication that must be considered is the high rate of muscular atrophy of the iliopsoas which can highly impact athlete’s performance [9].

**Table II.** Results and complications

*Study Authors (year)*	*Results and conclusions*	*Recurrence/complications*
Ilizaliturri et al. [2005] [14]	No snapping symptoms were present in any patient after surgery of at last follow up.	None
Flanum et al. [2007] [15]	None of the patients experienced recurrence of their snapping or pain	At 1-year follow-up 2 patients noted occasional slight pain in their hips.
Anderson and Keene [2008] [16]	All patients had resolution of snapping and all returned to sport on average 9 months after surgery	Six patients still experienced pain
Ilizaliturri et al. [2009] [17]	No differences were found between the 2 groups	None
Contreras et al. [2010] [18]	All patients returned to original or better level of function shortly after operation. All had maximum strength of hip flexion, extension, abduction, and adduction	Two patients had no improvement in pain despite resolution of the snapping. No patient had any post-operative complications.
Fabricant et al. [2012] [20]	The purpose of this study was to identify the functional outcomes of high version compared to low/normal version.	Patients with increased femoral anteversion may be at greater risk for inferior clinical outcomes after arthroscopic lengthening. No intraoperative or perioperative complications.
Hain et al. [2013] [21]	The majority of post-operative symptomatic patients have atrophy of the iliacus and psoas muscles and distortion and disruption of the iliopsoas tendon.	
Garala et al. [2014] [22]	Ten patients reported pain relief after their tenotomy and 5 patients reported no change in pain. For those patients with only temporary relief from injection, psoas tenotomy can provide good long-term pain relief.	In both groups of patients, exercise was the most affected category identified. Symptoms that patients complained of at 49 months after the tenotomy included pain (26%), stiffness (13%), instability (20%), decreased range of motion (20%) and snapping sensation (33%).
Ilizaliturri et al. [2014] [23]	Every patient in both groups had an improvement in WOMAC score.	One patient in group 2 presented with recurrence of snapping that required surgical intervention.
El Bitar et al. [2014] [24]	Statistically significant improvement in all PROs 81.8% good/excellent satisfaction and 81.8% resolution of painful snapping.	Revision surgery (*n* = 8; labral retear [*n* = 6], stiffness [*n* = 1], heterotopic ossification [*n* =1]), superficial wound infection (*n* = 1), perigenital numbness (*n* = 1)
Nelson and Keene [2014] [12]	An arthroscopic release of the iliopsoas tendon at the level of the labrum was effective for alleviating hip pain from labral lesions caused by impingement of the tendon in 23 of 30 patients (77%).	Recurrent snapping (*n* = 3) requiring iliopsoas bursa injections.Development of avascular necrosis (*n* = 1)Progression of degenerative joint disease (*n* = 1)Chronic greater trochanteric bursitis (*n* = 2)
Hwang et al. [2015] [5]	Snapping sound disappeared in 24 out of 25. Improvement in Harris Hip Score Values	Revision surgery (*n* = 1) for painful snapping
Brandenburg et al. [2016] [26]	In the release group, the iliopsoas muscle of the surgical limb was significantly smaller and weaker in the seated position (both P<001) than the contralateral limb	Iliopsoas atrophy with 25% volume loss and a 19% reduction in seated hip flexion strength in (25.3% of IFL group)
Mardones et al. [2016] [27]	Statistically significant improvement in patients functional scores (mHHS and Vail Sport Test)	Recurrence of pain 1-year post-operatively (*n* = 2)
Walczak et al. [2017] [9]	A majority of patients (89%) developed iliopsoas (IP) muscle atrophy after labral level IP tenotomies. The lesser trochanteric IP tenotomies did not develop atrophy of the gluteus maxims (*n* = 1) and vastus lateralis muscles, have chronic IP tendon disruption (*n* = 2), or develop the severity of IP atrophy (*n* = 3).	Iliopsoas tendon tear (*n* = 2), gluteal tendon tear (*n* = 1), lateral femoral cutaneous nerve injury (*n* = 1)
Hartigan et al. [2018] [28]	Patients with an LCEA of less than 25 and associated painful iliopsoas snapping can be treated by central-compartment IFL and have high satisfaction, improvement in PROs, and improved pain scores without significant progression of osteoarthritis.	Revision (*n* = 4) for traumatic labral retear, no complications
Perets et al. [2018] [6]	All PRO scores demonstrated significant improvements at latest follow-up (*P* < 0.001). Mean satisfaction was 7.9. No patients converted to arthroplasty. Painful snapping was resolved in 55 athletes (91.7%)	Temporary numbness (*n* = 1)
Maldonado et al. [2018] [8]	The IFL group showed comparable results to the control group with respect to PRO improvement.	Revision surgery (*n* = 17 in IFL group) and (*n* = 11 in non-IFL group); conversion to THA (*n* = 4 in IFL group) and (*n* = 7 in non-IFL group)
Perets et al. [2019] [7]	IFL as part of hip arthroscopy for treatment of FAI and labral tears demonstrated similar favorable improvement, complication rates, and secondary surgeries when compared with a control group that did not undergo IFL	Ten hips (17.5%) required secondary arthroscopy. Three hips (5.3%) required total hip arthroplasty. One case (1.8%) had minor post-operative complications
Meghpara et al. [2020] [4]	Both groups experienced significant improvements from pre-surgery to latest follow-up for all recorded PROs. The IFL group compared favorably with the control group for mHHS (86.0 versus 86.1; *P* = 0.53), NAHS (83.0 versus 84.7; *P* = 0.40), and HOSSSS (78.1 versus 76.5; *P* = 0.87). Additionally, iHOT-12, VAS, patient satisfaction, and rates of achieving the minimal clinically important difference for mHHS, NAHS, and HOS-SSS were similar between groups at the latest follow-up.	Study group (IFL): one hip required revision arthroscopy for labral tear and 2 hips converted to THA. 13 hips will persistent PISControl group (non-IFL) 1 hip required revision arthroscopy because of residual FAI
Maldonado et al. [2021] [8]	All patients in the study group demonstrated statistically significant improvement from pre-operative to latest follow-up in mHHS, NAHS, HOS-SSS, and VAS scores. Fifty-seven (78.1%) patients achieved or exceeded the minimal clinical important difference (MCID) for mHHS. For HOS-SSS 68.1% met or surpassed the MCID.	Study group 2 secondary arthroscopy and 1 total hip arthroplastyControl group 1 secondary arthroscopy and 1 total hip arthroplasty
Matsuda et al. [2021] [10]	Co-afflicted patients treated without tenotomy have similar successful outcomes to patients with primary FAI.	Co-afflicted patients with iliopsoas pathology treated with tenotomy had poorer outcomes compared with controls with FAI without iliopsoas pathology
Jimenez et al. [2022] [5]	89.5% of athletes who attempted to return to sport in IFL were successful. 76.0% of athletes who attempted to return in the non-IFL were successful. e main finding of the present study was that at minimum 5-year follow-up, competitive athletes who underwent primary hip arthroscopy for FAIS and IFL for painful internal snapping hip demonstrated significant improvement in all recorded PROs.	The IFL group underwent 2 revision arthroscopiesThe control group underwent 3 revision arthroscopiesControl group had higher rates of undergoing femorplasties when compared to the IFL group.

While these studies are essential to consider the possible negative outcomes of IFL, the methodology should be closely analyzed to fully interpret their findings. When analyzing the Meghpara study methodology, the results should only be used to answer the question of how to manage benign iliopsoas impingement, as described by Domb *et al*. in 2011 [12]. In their methodology, the control group that did not undergo an IFL did not have any pain. Therefore, this study should not be used to compare the overall effectiveness of IFL. Instead, this study should be used to conclude that patients without pain should not be considered good candidates for IFL.

The Matsuda study made even bolder claims against IFL. The tenotomy group (*n* = 16) was significantly smaller than the iliopsoas group without tenotomy (*n* = 76) [10]. There could have been selection bias for the tenotomy group, and their pathology could have been worse pre-operatively. The only PRO outcome was iHOT, while many of the studies in this review also included additional PRO such as mHHS, VAS and HOS. While there may be weaknesses in their methodology, both studies clearly show the importance of the patient selection process for success. To achieve optimal patient outcomes, surgeons should carefully assess patients’ suitability for the procedure based on individual needs and circumstances.

Regarding the most agreed-upon technique to treat ISHS, the literature does not state a superior technique. Several studies have used the central compartment technique and lesser trochanter technique, but none of the studies found a statistical difference in PRO. Further randomized clinical trials would be beneficial in this area.

As hip arthroscopy becomes increasingly popular and commonly used, additional techniques may be added to the procedures. Specifically, IFL has been shown to have positive PRO outcomes. Recently, these positive outcomes have been called into question which is exemplified by none of the comparative studies showing a significant improvement compared to the control non-IFL group. These new studies align with the trends reported in the paper by Chen *et al*. In their study, they reported that 75% of surgeons indicated a decrease in frequency of IFL. This is understandable because the IFL does have risks such as hip flexion weakness [9, 13].

Future research endeavors should prioritize investigating the complications linked to Iliopsoas Impingement (IFL). Additionally, forthcoming studies should aim to establish diagnostic methods that can distinguish between hip pain stemming from the iliopsoas muscle and that originating from the hip joint itself. To date, no studies have reported cases of persistent painful snapping hip after successfully addressing hip joint issues and resolving Femoroacetabular Impingement (FAI). Hence, it is advisable to address painful snapping hip through a two-stage approach. Initially, surgical correction of the hip joint should be pursued. If persistent pain remains, a subsequent surgery involving Iliopsoas Lengthening (IFL) may be considered.

## LIMITATIONS

Limitations of this study include the lack of high-quality evidence, primarily relying on retrospective studies with only one randomized controlled trial. Potential publication bias arises from excluding unpublished data, which may affect the comprehensiveness of the review. The varying follow-up durations hinder the assessment of long-term outcomes and complications. Heterogeneous outcome measures make comparisons challenging. Confounding factors and the absence of long-term complications data were not adequately addressed. The study’s generalizability is limited due to the specific populations included in the analysis. These limitations should be considered when interpreting the findings and applying them to clinical practice.

## CONCLUSION

Based on the available evidence, it is unclear if IFL provides any additional benefits to patients. Furthermore, there are inherent risks associated with the procedure, such as the potential for iliopsoas muscle atrophy. Therefore, the preferred treatment approach for individuals with internal snapping hip syndrome should prioritize addressing the underlying hip joint issue.

However, if the iliopsoas muscle is indeed responsible for the pain, there might be a role for IFL. Many of the studies in this review showed positive outcomes, including improved patient-reported assessments, pain relief, cessation of snapping sensations, and enhanced functionality across various patient populations. It's crucial to acknowledge, though, that these improvements cannot be solely attributed to IFL alone, as concurrent arthroscopic treatment for coexisting hip pathologies was also performed.

## DATA AVAIILABILITY

A total of 24 studies met the inclusion criteria and were included in the systematic review. The full-text articles for the included studies were obtained through institutional subscriptions, and the data extracted for analysis were reported in the review. No additional data beyond what is reported in the review is available.
